# Nitrite and Nitrate Concentrations in the Drinking Groundwater of Shiraz City, South-central Iran by Statistical Models

**Published:** 2017-09

**Authors:** Ahmad BADEE NEZHAD, Mohammad Mahdi EMAMJOMEH, Mahdi FARZADKIA, Ahmad JONIDI JAFARI, Mehrab SAYADI, Amir Hossein DAVOUDIAN TALAB

**Affiliations:** 1. Dept. of Environmental Health Engineering, School of Allied Medicine, Behbahan Faculty of Medical Sciences, Behbahan, Iran; 2. Dept. of Environmental Health Engineering, Social Determinant of Health Research Center, Qazvin University of Medical Sciences, Qazvin, Iran; 3. Dept. of Environmental Health Engineering, School of Public Health, Iran University of Medical Sciences, Tehran, Iran; 4. Research Center for Environmental Health Technology School of Public Health, Iran University of Medical Sciences, Tehran, Iran; 5. Research Committee, Shiraz University of Medical Sciences, Shiraz, Iran; 6. Dept. of Occupational Health, Behbahan Faculty of Medical Sciences, Behbahan, Iran

**Keywords:** Nitrite and nitrate, Drinking groundwater, Iran

## Abstract

**Background:**

Nitrite (NO_2−_) and nitrate (NO_3−_) contaminations of groundwater are considered as one of the major health challenges in recent decades. This study aimed to evaluate the nitrite and nitrate concentrations in the drinking groundwater of Shiraz, South-central Iran by statistical models.

**Methods:**

From 43 active wells of Shiraz drinking water supplies, 344 samples were collected in the high and low precipitation seasons from 2010 to 2014. Nitrate and nitrite concentrations were tested by a DR6000 spectrophotometer, and the results were analyzed by different software, including SPSS ver. 20, ArcView GIS 9.3. It was done by variable and multivariate regression models. In all tests, the level of significance was set at 5%.

**Results:**

Nitrate concentrations in the samples were in the range of 5 to 72 mg/l, and 38 (11%) of the samples had nitrate concentrations above the standard level 10 mg/L as nitrogen. The annual mean concentration of nitrite varied from 0 to 0.025 mg/l. There was an inverse correlation between nitrate and nitrite concentrations and well depths.

**Conclusion:**

The most important reason for the high concentration of nitrate in Shiraz drinking groundwater supplies were lack of health privacy of wells, the impact of residential construction around drinking water wells, and placement of wells in the direction of groundwater flow.

## Introduction

Groundwater resources are considered as one of the best and most important resources for drinking water (1, 2). Some materials have been reported as contaminants in the groundwater, including hydrocarbons, synthetic organic chemicals, mineral cations, mineral anions, pathogens, and radionuclide. Nitrate and nitrite are naturally happening ions that are part of the nitrogen cycle (3). Nitrate is in solution form and is changeable and a chemical factor that can affect health, potable water and can cause unfavorable health effects in consumers (4, 5).

Water pollution to nitrates can be arrived into water resources by water pollution of industrial and municipal waste, animal manure and fertilizer or industrial and municipal wastewater (6). When water passes through layers of the earth, it may enter to groundwater resources because of pollution of water with organic materials, decomposition of urban and industrial waste in the soil, washing of animal and chemical manures caused by agricultural activities as well as leakage of sewage (7, 8). What is certain is that nitrate in water may have undesirable effects such as carcinogenicity, congenital birth defects, cardiovascular diseases, blood pressure and the effect on the nervous systems (9, 10). Therefore, WHO has offered guidelines in this field. Consequently (11), the Institute of Standards & Industrial Research of Iran (SIRI) has attempted to determine the maximum allowable concentration (standard) of nitrate and nitrite in the drinking water to a level of 50 and 3 mg/l, respectively (12).

Statistical techniques have the ability to determine the concentration of nitrate. One of the statistical methods commonly used to predict the quality of groundwaters is Tobit regression models. It is a multivariate analytical model that can cause the relationship between the quality of ground waters and the other variables (13).

A study used the regression model and neural networks for predicting the concentration of nitrate in groundwater, showed the lack of significant differences between the projected concentrations of nitrate and its real concentration (14). Using multivariate regression Nolan showed that the maximum concentration of nitrate (nitrogen) is caused by population density and the application of fertilizers (15). There is a direct and significant relationship between concentrations of nitrate in groundwater with the land use; in addition, this model provided a reliable prediction of nitrate concentrations in groundwater resources (16). In some studies (17, 18), generally, the impact of land use on the concentration of nitrate has been investigated and other studies have investigated other aspects, including the impact of the development of urbanization, the passage of the groundwater, the amount of water and water table on nitrate concentration of groundwater sources.

This study aimed to investigate the nitrite and nitrate concentrations in Shiraz city drinking groundwater sources, and its main factors, by using variable and multivariate regression models.

## Methods

The study region was analytical, cross-sectional. In order to investigate the changes in concentration of nitrite and nitrate of underground sources of drinking water, 43 wells in Shiraz, City South-central Iran were studied. The samples were taken from each of these wells in a high and low precipitation seasons separately per year. Overall, 344 samples were regularly taken from 2010 to 2014. Totally 15, 14, and 8 wells located in the North, West, East and South East of the plain of Shiraz, respectively; and six other wells in the center of the plain of Shiraz, generally in urban texture. The amounts of Nitrite and Nitrate were measured by using a UV DR 6000 spectrophotometer with a length of 220nm, Hach model made in the USA (Standard Method c4500) and spectrophotometer (Visible) *Hach* model made in USA (Standard Method c4500).

### Working with geographic information system (GIS)

Using global positioning devices (GPS), UTM coordinates of the studied 43 wells were determined. Features of wells, including depth, name, the water table, texture and material, address and exact location, water yield, and chemically measured data in a table had been annexed to the above map. The zoning map of nitrate concentration in both times of dry (low precipitation) and wet (high precipitation) on the plain of Shiraz was drawn to determine the location of the concentrations of nitrate.

### Statistical tests

To determine the process of changes of the concentration of nitrate in the past and predicting it in the upcoming years, the statistical model of time series was used. Firstly, to determine the relationship between measured years and the amount of nitrate, the simple linear regression was used. In the next step, the correlation between these variables with the concentration of nitrates was determined and the *t*-test was used for the comparing these variables. Finally, in a multiple linear regression analysis, the relationship between nitrates with the parameters measured at the level of a significant 5% was calculated and the results were analyzed using SPSS version 18(Chicago, IL, USA)

## Results

**Fig. 1:**
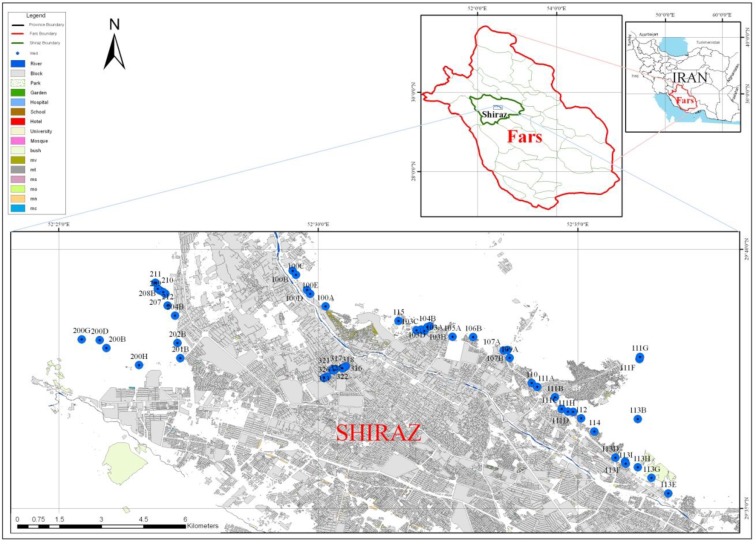
The area and the location of the wells studied

The area and the location of the wells studied are shown in Fig. 1. In the Western region, the wells had the lowest concentration of the average nitrate, while the highest average concentration of nitrate was found in the central region. Ten wells at least once had the concentration of nitrate more than the maximum limited concentration (the maximum range allowed Iran’s standard of 50 mg/l). In total, 38 samples (11%) were higher than the allowed standard (Table 1). The obtained results showed that the NO_2−_ concentration was lower than the amount of standard recommended in all samples. The mean, minimum and maximum of nitrite concentrations in studied wells were found to be 0.6, 0 and 0.025 mg/l, respectively.

**Table 1:** Frequency of minimum and maximum amount of nitrate in wells of drinking water in Shiraz City, South-central Iran

**Group of the wells**	**No. of Wells**	**Min of nitrate conc. (mg/l)**	**Max of nitrate conc. (mg/l)**	**Mean (mg/l)**	**No. of samples No_3_ con. exceeding the threshold (%)**	**No. of samples No_2−_con. exceeding the threshold**	**Max of nitrite conc. (mg/l)**	**Min of nitrite conc. (mg/l)**
**The wells of North**	15	11	51.2	24.5±15.04	5(1.45)	0	0.02	0
**The wells of East**	14	12	64	40.95±16.8	24(7)	0	0.013	0
**The wells of West**	8	5	12	6.56±2.43	0(0)	0	0.002	0
**The wells of Center**	6	34	71	44.88±8.97	10(2.9)	0	0.052	0

Based on the above table, the relationship between the average concentration of nitrate with geographic direction and location of collecting samples was obtained based on *t*-test. According to the results obtained using *t*-test, there was a significant difference between the amount of nitrate in a different direction (*P*<0.001). The amount of nitrate in different directions were not the same, this means that the average concentration of nitrate in the central wells of Shiraz Plain was a higher average. To determine the relationship between the measured years and the amount of nitrate a simple linear regression was used. No significant relationship existed between nitrates and measured years. The results of this analysis are summarized in Table 2.

**Table 2:** Analysis of regression variance of nitrate concentration changes relative to the time of the year

**Variable**	**B**	**SE**	**Beta**	**T**	***P* value**	**F**	**R^2^**
**Year**	−0.063	0.435	−0.009	0.145	0.855	0.021	0.001
**Constant**	3.33	1.73		17.5	<0.001		

**Fig. 2:**
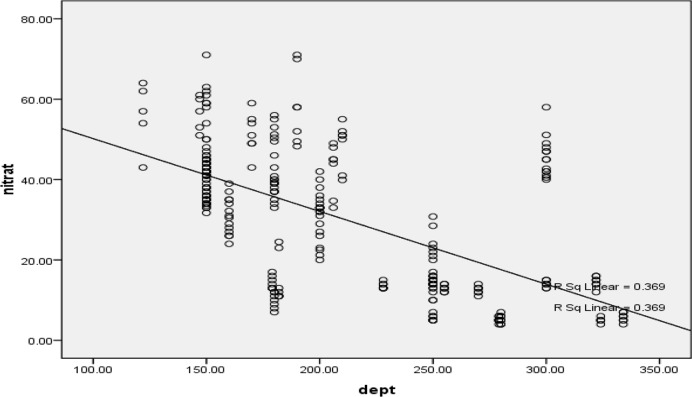
The relationship between the depth of the wells and the concentration of nitrate in groundwater drinking resources in Shiraz City, South-central Iran

**Fig. 3:**
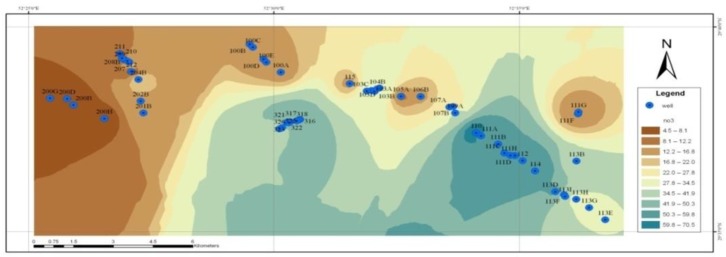
Zoning map of concentrations of nitrate in the high precipitation season

**Fig. 4:**
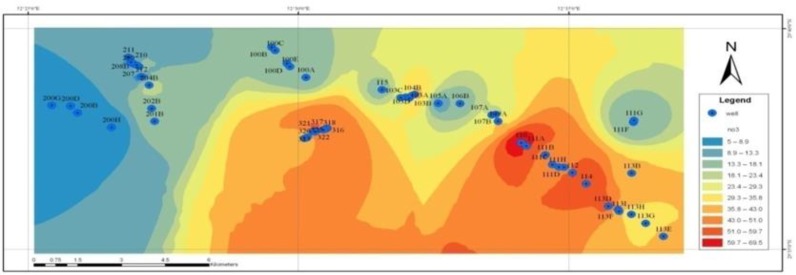
Zoning map of concentrations of nitrate in the low precipitation season

The relationship between the depth of the wells and the concentration of nitrate in drinking groundwater resources in Shiraz is shown in Fig. 2. It shows a zoning map of the concentration of nitrate in high precipitation season. Fig. 3, also shows the concentration of nitrate in low precipitation season. A comparison between these two figures showed that there was no difference between the concentration of nitrate in high and low precipitation seasons. Based on Fig. 4, the concentration of nitrite ion in water in the wells studied was nearly zero or very lower than the proposed standard values.

Comparison between the average concentrations of Nitrate with variables measured by concentration of nitrate is summarized in Table 3. The *t*-test was used for comparison between the variables. Except in variable adjacent to the dry river (*P*=0.834), there was a significant relationship between the amount of nitrate and the variable determining the status of wells (*P*<0.001). Based on this table, the average concentration of nitrate in wells without the health privacy was 47.62mg/l and in wells located within the scope of the radius of 100 meters in the residential context, compared with wells without these conditions, the average concentration of nitrates was more than twice.

**Table 3:** Comparison between the average concentrations of nitrate with variables measured by concentration of nitrate

**Variable**	**NO=0**	**YES=1**	***t***	***P*-value**
	47.62±8.74	24.09±15.28	12.34	<0.001
**Favorable Radius**				
**Health wells**				
**Residential land use**	18.23±11.33	46.33±8.8	22.77	<0.001
**Green lawn**	31.04±17.57	18.61±6.57	3.21	0.001
**Close to the sewage collection network**	41.95±17.86	36.03±11.88	2.44	0.016
**Located in the direction of ground water flow**	21.05±14.06	45.33±10.15	15.70	<0.001
**Distance from a dry river**	30.15±17.89	30.01±17.16	0.209	0.834

Finally, in a multiple linear regression analysis, the relationship between nitrates with the parameters measured at the significance level of 5% was calculated (Table 4). Based on the results, the multivariate regression linear equation was obtained, as follows:

$$y=43.05-0.076x_{1}+19.28x_{2}-4.45x_{3}+4.35x_{4}-11.07x_{5}-8.56x_{6}$$

Where y is the amount of nitrate and χ respectively from 1 to 6 including health radius compliance, residential use, agricultural use, sewage collection system, to be located in the direction of underground water flow and distance from a dry river.

Based on this model, there was a positive correlation between the variables of residential use in the wellhead protection area, location of wells compared to the path of the groundwater flow with concentrations of nitrate; moreover, there was a reverse relationship between the depths of the wells with concentration of nitrate in groundwater resources.

Based on the regression analysis model, there was relationship between nitrate concentration and the depth of the wells, residential privacy status, status of wastewater, and water flow path. This model also showed that there was no relationship between the concentrations of nitrate and time differences.

## Discussion

The results obtained suggest that a large amount of the study area and wells were affected by human activities and have been polluted. Based on Table 1, the average concentration of nitrate in the central wells of Shiraz plain is higher than wells in other areas. These wells are located in the city center and have an alluvial texture. The highest concentration of nitrates in these areas is due to the construction and urban development in these areas and the specific nature of the alluvial textures.

In the western region of Shiraz plain, wells have had the lowest concentration of nitrate among of all wells studied and the process of their changes relative to the time during the past few years is not sensible. The wells have been located in the most western part of the plain and in the foothills higher than the urban texture of Shiraz. The proper distance of the wells from residential texture and the lack of other polluting sources are the most important reasons for the very low concentrations of nitrate in the wells, although the three wells had nitrate concentrations higher than the rest of the wells. On many of the groundwater is caused by domestic sewages. For example, In the Suffolk region, Island in 2003 the concentration of nitrate was measured, the concentration of nitrate in the 43% of samples is more than 50 mg/l that this exceeds the acceptable limit of nitrate in drinking water. Ultimately, the main reasons for the high rate of nitrate in groundwater of these areas have been domestic wastewater and the high density of population (19).

Wells of the Eastern and Southeastern region are dispersed in the metropolitan area or have been located outside the metropolitan area; five wells are located in the urban context and the lack of health privacy at least 100 meters. These wells are located within the scope of the textures with the residential use.

Sewage systems were not favorably developed in these areas. On the other hand, these wells are located in plunge of surface slope of the North and East of the area to the South East of the Shiraz Plain, given that ground waters of the urban developed areas, affected, are located in this path; so, residential context, the lack of health privacy and to be located in the path of the underground aquifer flow (given that path of the movement of groundwater matches the direction of the slope of the Earth’s surface) has been effective in increasing high concentrations of nitrates in these areas.

According to Tables 2 and Fig. 2, a simple linear significant relationship was obtained between the depth of the well and the amount of nitrate and vice versa. This reduction of the nitrate concentration of nitrates simultaneously with an increase in the depth of the wells, suggests that the origin of the nitrate is mainly on the surface and sub-surface layers and by increasing the depth of the soil layer during penetration, the present salts remain in the upper stratum of soil; so, it is consistent with the results of studies conducted in China, Texas in the USA and South Korea (20–22).

According to Tables 3 and 4, wells that were lacking in health privacy, and wells located at 100 meters radius of the densest residential textures and the wells located at path of motion of groundwaters, have the highest average concentration of nitrate. Since the wastewater produced in the residential areas is excreted through absorption wells and thus, the wastewater produced enter in the closest to the aquifer and the groundwater table of underground water, and because the aquifer is mainly made of lime, so, sewage contamination through the seams in the aquifer and the lack of enough soil layers as a filter to remove pollutants led to increased nitrates in the wells. The movement of groundwater is affected by topography and the gradient of the ground and is usually in accordance with the direction of surface water movement. Today, discharge of wastewater into the wells is a major factor to the pollution of groundwater in urban areas.

**Table 4:** Regression analysis of factors and parameters affecting on the concentration of nitrate in drinking water resources of Shiraz City, Iran

**Variable**	**Unstandardized Coefficients**		**Standardized Coefficients Beta**	***t***	***P* value**	**F**	**R^2^**
	**B**	**Std. Error**					
**(Constant)**	43.056	43.056	0.004	10.946	<0.0001		
**Time in years**	0.03	0.03	−.255	0.166	0.868		
**Dept**	−0.076	−0.076	0.07	−7.382	<0.0001		
**Health radius compliance**	2.782	2.782	0.551	1.394	0.164		
**Residential use**	19.283	19.283	−0.041	8.358	<0.0001		
**Agricultural use**	−2.751	−2.751	−0.190	−1.427	0.155	128.42	0.837
**Sewage collection system**	−4.451	−4.451	0.122	−5.502	<0.001		
**Located in the direction of ground water flow**	4.355	4.355	−0.044	2.874	0.004		
**Distance from a dry river**	−1.779	−1.779	0.058	−1.192	0.234		

In recent years, due to the intensified or completed investigations the construction of a sewage collection system, especially in the central areas and areas where have wells with lower quality and without health privacy, the concentration of nitrate in these resources has had less increase.

The nitrate concentration zoning maps (Fig. 3 and 4) showed there is no considerable difference between amount of this anion in the two seasons of high and low precipitation. Since the outer surface of the plain is formed by a generally impermeable urban texture, it may be due to the lack of contamination of the nitrates in the direction of feeding the underground resources of Shiraz, because if they were to be together, too, the rainfall leads to leaching of nitrate and increased arrival of nitrate to the underground sources caused by concentration changes of nitrate in high precipitation seasons. In fact, rain has had not much impact directly in feeding the plain and become diluted concentrations of nitrate and seasonal variations. A similar study in the same direction around the Zayandehrud in Isfahan, in an annual period, showed the highest nitrate concentrations in groundwater have been obtained during the period of five months of Jan, Feb, March, Apr, and May. This topic may be due to consecutive leaching of nitrate from the soil with rainfall in the winter season and coincides with the added fertilizers to the soil (23). In other studies (24, 25), from this perspective, they are inconsistent with the present study. Perhaps, because the surface of Shiraz plain is formed by the impermeable urban texture and as a result, feeding groundwater is not consistent with nitrate pollution origin.

**Fig. 5:**
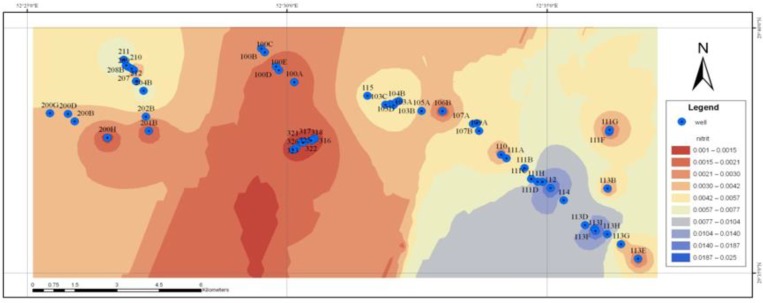
Zoning map of concentrations of nitrites

Based on the zoning map, the values of nitrite ion in water of the studied wells was nearly zero or very lower than the proposed standard values (Fig. 5). This is the result of being unstable nitrite and its rapid conversion to nitrate ions in nature. The high concentrations of nitrate ion, at the same time, low concentrations of nitrite ion, shows the fact that the existing pollution did not happen recently and for a moment, but this increase occurred during many years.

## Conclusion

The value of the coefficient of determination in a regression equation was 83.7% that is statistically a great extent and this means that almost 84% changes of nitrate are justified using the mentioned variables. In the western areas of Shiraz Plain, wells have best quality, and in central areas of Shiraz plain, they have highest average concentration of nitrate. The most important reason of the high concentrations of nitrate in wells in Shiraz plain is lack of health privacy of wells and the impact of the residential use around the wells; in addition, placement of wells on the groundwater motion path was another factor affecting the high concentration of nitrates. Over the years the concentration of nitrate has had the incremental trends and in some of the wells led to the rise higher than standards, though in the last few years, with the development of the sewage collection system, speed in the increase of nitrate is somewhat controlled.

## Ethical considerations

Ethical issues (Including plagiarism, informed consent, misconduct, data fabrication and/or falsification, double publication and/or submission, redundancy, etc.) have been completely observed by the authors.
